# *Little ROCK *is a *ROCK1 *pseudogene expressed in human smooth muscle cells

**DOI:** 10.1186/1471-2156-11-22

**Published:** 2010-04-14

**Authors:** Maria Claudia Montefusco, Kristen Merlo, Crystal D Bryan, Howard K Surks, Steven E Reis, Michael E Mendelsohn, Gordon S Huggins

**Affiliations:** 1 Molecular Cardiology Research Institute, Tufts Medical Center, 800 Washington Street, Boston 02111, MA, USA; 2 University of Pittsburgh, 3550 Terrace Street Pittsburgh, PA 15261, USA

## Abstract

**Background:**

Sequencing of the human genome has identified numerous chromosome copy number additions and subtractions that include stable partial gene duplications and pseudogenes that when not properly annotated can interfere with genetic analysis. As an example of this problem, an evolutionary chromosome event in the primate ancestral chromosome 18 produced a partial duplication and inversion of rho-associated protein kinase 1 (*ROCK1 *-18q11.1, 33 exons) in the subtelomeric region of the p arm of chromosome 18 detectable only in humans. *ROCK1 *and the partial gene copy, which the gene databases also currently call *ROCK1*, include non-unique single nucleotide polymorphisms (SNPs).

**Results:**

Here, we characterize this partial gene copy of the human *ROCK1*, termed *Little ROCK*, located at 18p11.32. *Little ROCK *includes five exons, four of which share 99% identity with the terminal four exons of *ROCK1 *and one of which is unique to *Little ROCK*. In human while *ROCK1 *is expressed in many organs, *Little ROCK *expression is restricted to vascular smooth muscle cell (VSMC) lines and organs rich in smooth muscle. The single nucleotide polymorphism database (dbSNP) lists multiple variants contained in the region shared by *ROCK1 *and *Little ROCK*. Using gene and cDNA sequence analysis we clarified the origins of two non-synonymous SNPs annotated in the genome to actually be fixed differences between the *ROCK1 *and the *Little ROCK *gene sequences. Two additional coding SNPs were valid polymorphisms selectively within *Little ROCK*. Little ROCK-Green Fluorescent fusion proteins were highly unstable and degraded by the ubiquitin-proteasome system *in vitro*.

**Conclusion:**

In this report we have characterized *Little ROCK *(*ROCK1P1*), a human expressed pseudogene derived from partial duplication of *ROCK1*. The large number of pseudogenes in the human genome creates significant genetic diversity. Our findings emphasize the importance of taking into consideration pseudogenes in all candidate gene and genome-wide association studies, as well as the need for complete annotation of human pseudogenome.

## Background

The ROCK1 and 2 serine/threonine kinases regulate many cellular responses such as cell growth, proliferation, and apoptosis through their effects on the cytoskeleton and microtubule network organization [1,2]. The ROCK1 and ROCK2 proteins share a similar structure characterized by an amino terminal coiled-coil domain containing the kinase activity, a Rho binding site, and a carboxy-terminal pleckstrin homology (PH) domain [3]. Activation by GTP-bound Rho-A involves displacement of the PH domain and exposure of the kinase domain to substrate [4-8]. In vascular smooth muscle cells (VSMC) ROCK1 and 2 activity promotes cellular contraction by direct phosphorylation of the myosin binding subunit (MBS) leading to inhibition of myosin light chain phosphatase activity [9,10]. Activated Rho kinases can also trigger phosphorylation of MBS through the Zip-like kinase [11,12] or by phosphorylating the CPI-17 protein, which physically binds and inhibits the actions of PP1M, the catalytic subunit of MLCP [13,14]. VSMC contraction triggered by activation of the ROCK1 and ROCK2 pathway causes blood vessels to constrict, which increases blood pressure [15]. Inhibitors of ROCK1 and 2 block VSMC contraction and lower blood pressure (BP) in humans [16], block acetylcholine-induced arterial vasoconstriction [17], and improve exercise-induced myocardial ischemia [18].

Given the importance of ROCK1 and ROCK2 to BP and by extension cardiovascular diseases we sought to understand whether genetic differences in these genes contribute to the normal variation of blood pressure that exists in the general population. The ROCK1 and ROCK2 proteins are products of separate genes located on chromosomes 18 and 2, respectively. A *ROCK2 *gene polymorphism located adjacent to the coiled-coiled domain (ROCK2-T432N) has been associated with BP [19]. At the start of our study computational analysis of *ROCK1 *gene revealed that the single nucleotide polymorphism database (dbSNP) lists several *ROCK1 *coding region variants, assigned to two different loci on chromosome 18. Reported studies designed to determine the genomic differences that distinguish the human chromosome 18 from its homolog in great apes (chimpanzee, orangutan, and gorilla) identified a chromosome 18 pericentric break causing an inversion and transposition event that included part of *ROCK1 *as well as *USP14 *and *THOC1 *[20,21]. The result of this chromosomal event, which occurred at some point before humans evolutionarily separated from great apes, was the placement of *USP14*, *THOC1 *and a partial duplication of *ROCK1 *in the sub-telomeric region of the p arm of chromosome 18 [20,21]. Full-length *ROCK1 *remained in the peri-centromeric region of 18q. This partial duplication corresponds to the region of *ROCK1 *(the last for exons and introns) that included numerous non-uniquely annotated coding SNPs.

Partial gene duplications commonly produce pseudogenes, and we considered whether the partial duplication of *ROCK1 *at 18p11.32 represented a *ROCK1 *pseudogene [22]. Approximately half of all mammalian protein families include pseudogenes http://pseudofam.pseudogene.org, with the greatest representation found in housekeeping and ribosomal families of genes [23]. While pseudogenes are commonly considered to be genetic "fossils" that have no biological function, there are examples of functional pseudogenes. Expressed pseudogene transcripts can contribute to the synthesis of small interfering RNA species that regulate parent transcripts [24,25], and disease-related pseudogenes have also been reported [26]. A pseudogene can be found for approximately twenty percent of kinase genes [27,28]; however a *ROCK1 *or *-2 *pseudogene has not been described. The microtubule-affinity regulating kinase family has the largest number of pseudogenes followed by p70S6 kinase [27]. Kinase pseudogenes that produce mRNA transcripts have been identified, but as yet there is no documented function for these expressed pseudogenes.

Here we report the characterization of the gene produced by partial duplication of human *ROCK1*, which we named *Little ROCK *(*ROCK1P1*). We demonstrate expression of Little ROCK transcript in human vascular smooth muscle cells. Despite the high level of nucleotide identity, we define sequences specific to *Little ROCK *and resolve the location of non-synonymous coding polymorphisms that were previously reported to be located non-uniquely within both *ROCK1 *and *Little ROCK*.

## Results

### *Little ROCK *is a partial duplication of the *ROCK1 *gene

**Figure 1:**
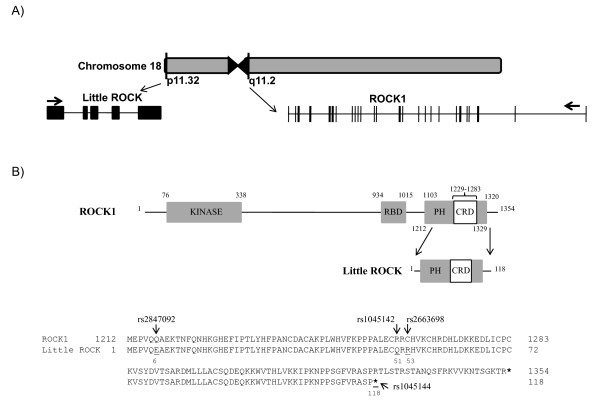
**Comparison of *Little ROCK *and *ROCK1 *genes**. (A) Cartoon demonstrates location of *Little ROCK *and *ROCK1 *on chromosome 18 and their exon gene structures. (B) The location of the *Little ROCK *coding sequence relative to *ROCK1 *is shown. ROCK1 includes a kinase domain and a Rho binding domain (RBD). Little ROCK and ROCK1 share pleckstrin homology (PH) and cysteine rich (CRD) domains. The amino acid sequence alignment and location of variants reported in dbSNP are shown.

While researching *ROCK1 *nucleotide coding polymorphisms we noted that dbSNP reported the location of several potential coding variants in two different parts of chromosome 18. Indeed, the Ensembl genome browser reported SNP entries on chromosome 18 both in the *ROCK1 *gene (ENSG00000067900) and in the partial duplication of *ROCK1 *(ENSG00000215585). For purposes of this report, we call the smaller partial duplication *Little ROCK*. Consistent with its derivation as a partial duplication and inversion event, *Little ROCK *maps to 18p11.32 in the sub-telomeric region, while *ROCK1 *is located in the opposite orientation in 18q11.1 near the centromer (Figure 1A). Compared with *ROCK1*, which has 33 exons and spans more than 150 kb, the *Little ROCK *gene spans about 13 kb and is predicted to include 5 exons. We compared the predicted nucleotide sequences of the two genes and found that the last 4 exons of both genes (exons 2 to 5 of *Little ROCK *and exons 30 to 33 for *ROCK1*) share 99% nucleotide identity. The predicted first *Little ROCK *exon is unique and not shared by *ROCK1*. The Little ROCK mRNA sequence is predicted to be 2497 bp, while the ROCK1 mRNA measures 6650 base pairs. Little ROCK transcript includes a potential initiator methionine residue in exon 2, and the predicted protein product is 118 amino acids. The predicted *Little ROCK *stop codon is located in the exon 4. Nucleotide BLAST studies did not find any *Little ROCK *homologues in other mammalian species, and we did not detect a duplication of *ROCK2 *in humans.

### Little ROCK transcript is detected in human VSMC and in human organs rich in smooth muscle cells

**Figure 2:**
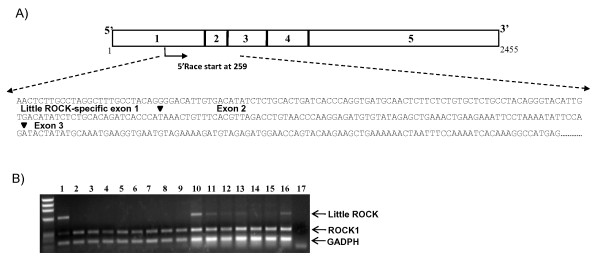
**Little ROCK Expression Analysis**. (A) Nucleotide sequence of a clone isolated by 5'RACE confirmed the Little ROCK transcript includes splicing of sequences with high identity to ROCK1 exon 30 to the Little ROCK-specific Exon 1. Arrowheads indicate exon splice sites. (B) Little ROCK, ROCK1 and GAPDH amplicons from cDNA produced from Lanes: 1-Testis, 2-Heart, 3-Kidney, 4-Brain, 5- Liver, 6- Lung, 7-Pancreas, 8-Placenta, and 9-Sk. Muscle. Lanes 10 to 16 are from cultured human VSMC lines: 10-IM230, 11-Ao146, 12-IM306, 13-Ao297, 14-IM337, 15-Co338, 16-Ao207. Lane 17 shows a negative control. GAPDH was used as a cDNA loading control.

We found a cDNA-derived sequence containing the Little ROCK-specific sequence including the predicted non-synonymous coding variants in the nucleotide database (BC041849). To confirm expression of *Little ROCK*, we performed 5' RACE analysis of human VSMC cDNA using reverse primers that anneal ROCK1 and Little ROCK. We isolated a total of 9 clones, one of which included the sequence that matched the Little ROCK-predicted exon at nucleotide 259 of the mRNA sequence reported by Ensembl genome browser (ENST00000400614) (Figure 2A). Having confirmed expression, we designed RT-PCR assays for analysis of Little ROCK and ROCK1 transcripts using cDNA obtained from cell lines and several human organs. We found the *Little ROCK *transcript in multiple cultured vascular smooth muscle cell lines and in testis, stomach, and vagina (Figure 2B). By comparison, *ROCK1 *was expressed in all organs and VSMC lines tested.

### *Little ROCK *has both fixed sequence differences compared with *ROCK1 *as well as non-synonymous polymorphisms

**Figure 3:**
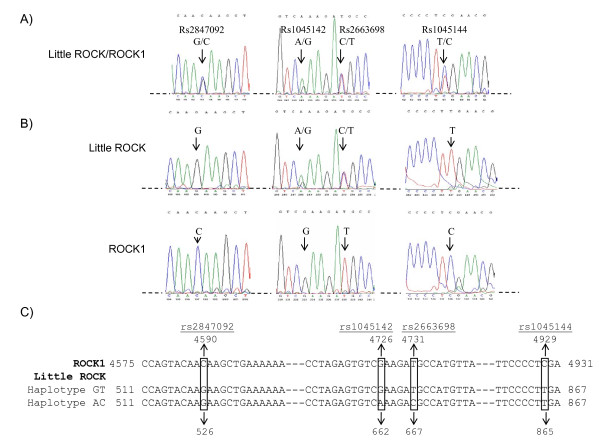
**Analysis of ROCK1/Little Rock nucleotide differences**. (A) Chromatograms from amplified genomic DNA that includes both ROCK1 and Little ROCK. Gene specific exon amplification was not possible due to high sequence identity. The location of variants identified in dbSNP is shown. Overlapping chromatogram peaks with similar sizes indicates locations of sequence variability. (B) Chromatograms from amplified cDNA. The use of a primer specific for the Little ROCK-specific exon and a primer specific to ROCK1 ensured the specificity of each amplicon. No ambiguous bases were found in the ROCK1 cDNA while rs1045142 and rs2663698 were heterozygous in the Little ROCK cDNA amplicon. (C) The location of fixed sequence differences created by ROCK1 and Little ROCK sequence differences (rs2847092 and rs1045144) and Little ROCK Haplotype AC and Haplotype GT created by linkage disequilibrium between polymorphisms rs1045142 and rs2663698 are shown. Sequence findings represent the results from 90 unique Caucasian DNA samples and from cultured VSMC cDNA analysis.

Comparison of the *Little ROCK *and the *ROCK1 *nucleotide sequences predicted several non-synonymous sequence variants located at Little ROCK mRNA positions C526G (rs2847092), A662G (rs1045142), C667T (rs2663698), and T865C (rs1045144). Because both the SNP database and the Ensembl Genome Browser could not resolve whether those variants were located in *ROCK1 *or *Little ROCK *we sought to characterize and to define the location of these sequence differences. The high level of intron and exon sequence identity between *Little ROCK *and *ROCK1 *prevented the development of assays that selectively amplify *ROCK1 *and *Little ROCK *exons from genomic DNA. By comparison, through the use of forward primers that recognize the *Little ROCK *exon 1 sequence or the *ROCK1 *exon 29 sequence we were able to specifically amplify the Little ROCK and ROCK1 cDNA sequences. With these assays we analyzed and compared amplicon sequence produced from genomic DNA (Figure 3A) and cDNA (Figure 3B)

C526G (rs2847092) and T865C (rs1045144) are located in the exon 3 and exon 4 of *Little ROCK *respectively corresponding to exon 31 and 32 of *ROCK1*. The DNA analysis of 90 unique human Caucasian DNA samples using a TaqMan assay for T865C and by direct sequencing of G526C showed heterozygote genotypes for all samples analyzed (Figure 3A). Sequence analysis of cDNA synthesized from human VSMC RNA revealed that G526 and T865 belong to Little ROCK (Figure 3B). From these results we conclude that both these nucleotide "variants" are actually fixed sequence differences between the *ROCK1 *and the *Little ROCK *genes.

A662G (rs1045142) and C667T (rs2663698) nucleotide variants are both within the exon 3 of *Little ROCK *corresponding to exon 31 of *ROCK1 *gene. The direct sequence analysis of 90 unique human Caucasian DNA samples showed polymorphic results where both homozygous GT and heterozygous GT and AC individuals were represented within the population. Sequence analysis of the VSMC ROCK1 cDNA sequence showed exclusively GT at the two positions (Figure 3B), which indicates that ROCK1 is not the source of these polymorphisms. By comparison, the Little ROCK cDNA sequence showed GT, AC or both. Sequence analysis further demonstrated that both polymorphisms are in complete linkage disequilibrium, forming two haplotypes: A662-C667 (Haplotype AC) and G662-T667 (Haplotype GT) (Figure 3C). To confirm the results and to determine the haplotype frequencies we genotyped the Heart SCORE cohort using a custom designed TaqMan assay. The frequency of the Haplotype AC was similar in Heart SCORE African American and Caucasian participants (minor allele frequency 0.33 versus 0.31, respectively). The two haplotypes were in Hardy-Weinberg equilibrium (p > 0.05) in both racial groups.

### Little ROCK transcript abundance is reduced compared with ROCK1

The custom Little ROCK/ROCK1 TaqMan assay can discriminate the T865C alleles in both genomic DNA and cDNA. Quantitative RT-PCR analysis of four unique human VSMC lines demonstrated that ROCK1 transcript had a lower threshold cycle for detection compared with Little ROCK (Ct mean ± SD 19.26 ± 0.68 versus 22.39 ± 1.29, p < 0.05), which is consistent with higher relative transcript abundance. By comparison, the *ROCK1 *threshold cycles were not lower than *Little ROCK *in analysis of genomic DNA from the same cell lines. Therefore, we conclude that while we confirmed expression of Little ROCK by 5'RACE and RT-PCR, its expression in smooth muscle cells is reduced compared with ROCK1.

### Little ROCK protein is degraded through a proteasome-dependent pathway

**Figure 4:**
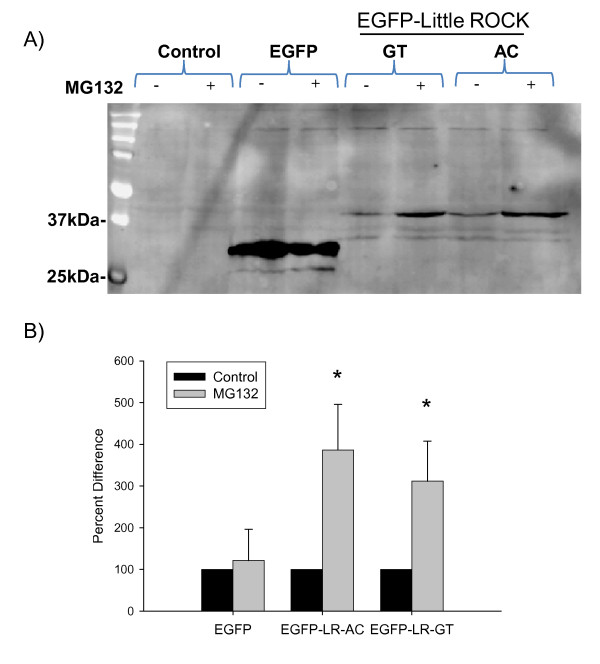
**Little ROCK1 Peptide is Unstable**. (A) Western blot of transfected HeLa cells probed with an antibody to GFP. Non-transfected control cell lysates are shown in lanes 1 and 2. Transfected cells were treated with MG132 (+) or control solvent (-). Expression of EGFP (lanes 3 and 4) was not affected by MG132 treatment. Cells expressing EGFP-LR-GT (lanes 5 and 6) and EGFP-LR-AC (lanes 7 and 8) included a slower migrating band consistent with the EGFP-Little Rock fusion peptide. MG132 treatment increased the abundance of the EGFP-Little ROCK fusion peptides. (B) Bar graph demonstrates mean ± SD intensity of peptide band normalized to treatment with solvent control. The experiment was performed three times; asterisk indicates the mean intensity of MG132-treated recombinant protein level was significantly different (*p *< 0.05) versus control treatment as determined by t-test analysis.

A characteristic feature of pseudogenes is their lack of a stable protein product [22]. We next tested whether the Little ROCK protein could be expressed. We cloned cDNAs encoding Little ROCK-Q51-R53 (Haplotype AC) or -R51-C53 (Haplotype GT) into expression vectors fused to a myc-epitope coding sequence. We were unable to demonstrate expression of N-terminal myc-epitope tagged Little ROCK in Cos7 and HeLa cells (data not shown). To provide a more stable chimera, we exchanged the myc-epitope for enhanced green fluorescent protein (EGFP) and expressed them in HeLa cells. Little ROCK-Q51-R53-EGFP and Little ROCK-R51-C53-EGFP fusion proteins migrated more slowly than EGFP due to the presence of Little ROCK. The increase in size of the fusion proteins was consistent with the addition of the predicted size of Little ROCK (13.5 kDa) to EGFP. Notably, the abundance of the Little ROCK-EGFP fusion proteins was significantly reduced compared with the control EGFP protein (90% less than EGFP). We next explored the possibility that Little ROCK is a target of the ubiquitin proteasome pathway that regulates the levels of damaged proteins within the cell. Treatment of transfected cells with the proteasome inhibitor MG132 [29] significantly increased the abundance of the fusion protein (Figure 4) demonstrating that Little ROCK destabilized EGFP in a proteasome-dependent manner. By comparison, EGFP was not significantly stabilized by MG132 suggesting that the effect of proteasome inhibition on the fusion protein was mediated through effects from the Little ROCK peptide sequence.

## Discussion

Completion of the human genome sequence has identified numerous chromosome copy number additions and subtractions that include partial gene duplication. Here we characterize *Little ROCK*, created by partial duplication and translocation of a portion of chromosome 18 immediately following the separation of humans from great apes [20,21,30]. Pseudogenes are one product of these duplication events, and several kinase pseudogenes have been described. We conclude that *Little ROCK *is a *ROCK1 *pseudogene for several reasons. First, despite a high degree of sequence identity with *ROCK1, Little ROCK *includes a disproportionate number of non-synonymous changes in the coding sequence. An excess of non-synonymous coding changes is characteristic of pseudogenes, perhaps reflecting a lower level of purifying selection as the parent gene [31]. Second, pseudogenes lack regulatory CpG islands in their promoters [22], and unlike *ROCK1 *and *ROCK2 *the first *Little ROCK *exon is not preceded by a predicted CpG island. We detected the Little ROCK transcript in cultured human VSMC, which is unusual because many pseudogenes are not expressed [22]. The expression pattern of Little ROCK transcript was different than ROCK1, a finding that likely reflected that the *ROCK1 *upstream gene regulatory promoter region was not included in the partial duplication event that created *Little ROCK*, and because *Little ROCK *is located near a telomere [20,21]. Finally, we found the Little ROCK protein to be highly unstable and capable of rendering a stable protein, EGFP, subject to degradation by the ubiquitin-proteasome system. Therefore, despite evidence of transcript expression, the highly unstable Little ROCK peptide is unlikely to accumulate to sufficient quantities to have a direct functional impact. Therefore, based upon the unique presence of a partial duplication of *ROCK1*, and the fact that a corresponding *ROCK2 *duplication was not found, our findings are consistent with *Little ROCK *being the sole pseudogene of the ROCK family of kinases. We have reported our findings to the HUGO Gene Nomenclature Committee and *Little ROCK *has been assigned the symbol: *ROCK1P1*.

Following completion of the human genome sequence and analysis of human gene variants it has been estimated that 12% of the human genome is affected by chromosomal gains and losses [32]. Indeed, an emerging problem is the incomplete annotation of stable chromosomal duplications and the pseudogenes often contained within the duplicated regions. Determining the location and sequence differences associated with chromosomal duplication is challenging because of the high level of sequence identity between pseudogenes and their parent genes. To illustrate this problem we have clarified the chromosomal location of four variants listed in SNP databases that at the time of preparing this manuscript were assigned both to *ROCK1 *and to *Little ROCK*. Two of these reported polymorphisms (rs2847092 and rs1045144) were in fact fixed nucleotide differences that define *Little ROCK *compared with *ROCK1*. By comparison we demonstrate two polymorphisms in complete linkage disequilibrium located exclusively in *Little ROCK *(rs1045142 and rs2663698). The polymorphic differences detected in Heart SCORE participants were found in similar allele frequencies in Caucasians and African Americans suggesting that the variants were created sometime after the time of the chromosome 18 event that created *Little ROCK *and before divergence of *Homo sapiens*. These examples illustrate the need to identify sequence differences of chromosomal duplications in the ongoing 1,000 Genomes Project.

Pseudogenes are commonly felt to be "junk" DNA, yet there are examples of pseudogenes that have functional regulatory effects [33,34]. As an expressed pseudogene, *Little ROCK *may also have a direct biological effect, perhaps affecting VSMC and blood vessel function. The instability of the Little ROCK fusion protein would suggest that any functional role may be unlikely to be explained on a peptide level. Future studies will explore the cross-talk between the Little ROCK and ROCK1 transcripts.

## Conclusion

In this report we have characterized *Little ROCK*, an expressed pseudogene derived from partial duplication of *ROCK1*. The large number of pseudogenes in the human genome creates significant genetic diversity that can have physiological importance. The finding of genetic variants distinct to *Little ROCK *emphasizes the importance of taking into consideration pseudogenes in all candidate gene and genome-wide association studies, as well as the need for complete annotation of human pseudogenome.

## Methods

### Genomic databases

Human *ROCK1 *and *Little ROCK *genomic, mRNA, and protein sequences were obtained from UCSC genome browser http://genome.ucsc.edu/cgi-bin/hgGateway and Ensembl genome browser http://www.ensembl.org/. In Ensembl genome browser, the identification numbers for *Little ROCK *and *ROCK1 *genes are ENSG00000215585 and ENSG00000067900, respectively.

### *Little ROCK/ROCK1 *DNA and cDNA analysis

Custom oligonucleotide primers designed manually were synthesized to amplify and sequence the shared *Little ROCK *and *ROCK1 *exons and nearby intron sequences as well as the cDNA sequences (Table 1). Primers LTEX3F and LTEX3R were used to amplify exon 3/exon 31 (product 437 bp) and primers LTEX4F and LTEX4R amplified exon 4/exon 32 (product 289 bp). To amplify Little ROCK and ROCK1 cDNA fragments including the nucleotide changes of interest, we designed 2 gene specific forward primers and 1 common reverse primer. The primers used for Little ROCK cDNA were cDNALTF and cDNACMR; for ROCK1 cDNA amplification we used cDNARKF and cDNACMR. The PCR products were obtained following standard PCR conditions with an initial step at 95° for 10 minutes followed by 40 cycles of three steps at 95° for 30 sec, 57° (exon 3 amplicon) or 60° (exon 4 and cDNAs amplicons) for 20 sec and 72° for 30 sec. A final step at 72° for 1 min was added to complete the elongation reactions. The PCR reaction was carried out in a final volume of 30 μl including 3 μl of cDNA (1/10 of the RT product), 1× polymerase reaction buffer (1.5 mM MgCl_2_), 0.1 mM dNTPs, 0.1 μM each primer, and 2 units of AmpliTaq DNA Polymerase (Applied Biosystems). After purification of the PCR product, 125 ng of amplicons and 5 pmoles of primers were used to carry out the sequencing reaction using Dye Terminator kit (Applied Biosystems, Foster City, CA).

**Table 1:** PCR, sequencing, site-directed mutagenesis primer and TaqMan probe Sequences

Assay	Name	Sequence
**PCR or Sequencing**	LTEX3F	5'-TTTTAAAGAATCTAAGTCCTAAGCG-3'
	LTEX3R	5'-TACACATAAGTTAGTTCATTGAGAC-3'
	LTEX4F	5'-CATCAGCAAGAGATATGCTGC-3'
	LTEX4R	5'-CCTCTGTGGTGAAAAGCACAA-3'
	cDNALTF	5'-TCTCTGCACAGATCACCCAGTAAACTG-3'
	cDNARKF	5'-GCTGGAAGAAACAGTATGTTGTGG-3'
	cDNACMR	5'-TAGCATCCCACACGATTCCAC-3'
	5RACELT	5'-CTTTGGCACAGGCATCACAATTGGC-3'
	ROCKF	5'-ATGGTACGATGTGATACAGCG-3'
	ROCKR	5'-CTCACTTCCCTGTCAGTAAGG-3'
	GAPDHF	5'-GTCGGAGTCAACGGATTTGGT-3'
	GAPDHR	5'-GCCATGGGTGGAATCATATTGG-3'
	LTGFPF	5'-CCGGAATTCGAACCAGTACAAGAAGCTGAA-3
		
TaqMan Assay	Rs1045144F	5'-TCCACCATCTGGTTTTGTTCGT-3'
	Rs1045144R	5'-CGGAAAGACTGATTTGCAGTGGAT-3'
	rs1045144-VIC	5'-TTCCCCTTGAACGCT-3'
	rs1045144-FAM	5'-CCCCTCGAACGCT-3'
	rs1045142/rs2663698F	5'-CCAAACCTCTCTGGCATGTTTT-3'
	rs1045142/rs2663698R	5'-CTTTCTTATCTAAGTGATCTCTGTGGCA-3'
	rs1045142/rs2663698-VIC	5'-CATGGCATCTTCGACACT-3'
	rs1045142/rs2663698-FAM	5'-ATGGCGTCTTTGACACT-3'
		
Mutagenesis	MU2LTR	5'-ACTTAACATGGCATCTTCGACACTCTAGGG-3'
	MU2LTF	5'-CCCTAGAGTGTCGAAGATGCCATGTTAAGT-3'

### Human Cohort DNA Studies

The Tufts Medical Center Institutional Review Board approved these studies. 90 human DNA samples collected from Caucasian patients with cardiovascular disease were used for gene sequencing studies. Heart Strategies Concentrating on Risk Evaluation (Heart SCORE) is a single-site prospective community-based cohort study investigating the mechanisms underlying population disparities in cardiovascular disease [35,36]. Our sample included 1,191 individuals (425 African Americans and 766 Caucasians) who provided consent and a DNA sample. Observed genotype frequencies were compared with those expected under Hardy-Weinberg equilibrium (HWE) using a χ^2 ^test.

### TaqMan genotyping assay

Custom TaqMan genotyping assays created for rs1045144 and the haplotype rs1045142/rs2663698 (See Table 1 for primer and probe sequences) were purchased from Applied Biosystems (Assays on Demand). The assays were performed following manufacturing instructions on a 7900HT real-time PCR system. The reaction volume was 5 μl and included 10 ng of DNA, 2.5 μl of Universal PCR master mix (2×) and 0.1 μl of 40× probes. The reaction conditions were: one step at 95° for 10 minutes followed by 40 cycles of 15 seconds at 92°C and 1 minute at 60°C. Real time PCR results and genotype calls were using the SDS 2.3 program (Applied Biosystems).

### Expression Analysis in Human Vascular Smooth Muscle Cell Lines

Immortalized human VSMC were provided by Dr. Mendelsohn. Details about explants, isolation, and immortalization of VSMC are reported in Pace MC *et. al*. [37]. VSMC cultures were maintained at 37°C in 5% CO_2 _humid atmosphere in a growth medium containing high glucose DMEM, Fetal Bovine Serum (10%), and Penicillin/Streptomycin (1×). Total RNA was extracted from VSMC using Trizol solution (Invitrogen, Carlsbad, CA) following the manufacturer's instructions. 5' RACE experiments were carried out on human VSMC RNA with a custom oligonucleotide primer that recognizes both ROCK1 and Little ROCK transcripts (5RACELT, Table 1) according to the GeneRacer kit instructions (Invitrogen). Due to the sequence similarity between *ROCK1 *and *Little ROCK*, one single gene-specific primer was designed in order to select both Little ROCK and ROCK1 mRNAs. RT experiments were carried out using SuperScriptIII enzyme (Invitrogen) on 5 μg of total RNA extracted from human VSMC. 1/10 of the RT reaction volume was used for the following PCR. A set of eight human organ cDNAs were purchased from Clontech (Clontech, Mountain View, CA). We carried out PCRs for *Little ROCK*, *ROCK1 *and *GADPH *cDNAs on all cDNA samples. GAPDH was use as a cDNA loading control. The *Little ROCK *primers were: cDNALTF and cDNACMR, resulting in a 614 bp PCR product; *ROCK1 *primers were: ROCKF and ROCKR resulting in a 230 bp PCR product; and GAPDH primers were: GAPDHF and GAPDHR, producing a 146 bp PCR product. Primer sequences are listed in Table 1. The PCR reaction was carried out in a final volume of 30 μl including 3 μl of cDNA, 1× polymerase reaction buffer (1.5 mM MgCl_2_), 0.1 mM dNTPs, 0.1 μM each primer, and 2 units of AmpliTaq DNA Polymerase (Applied Biosystems). Standard PCR conditions have been used: an initial step at 95° for 10 minutes followed by 35 cycles of three steps at 95° for 30 sec, 60° for 20 sec and 72° for 50 sec. A final step at 72° for 1 min was added to complete the elongation reactions.

### Cloning of *Little ROCK *cDNA

Little ROCK coding region was cloned into pEGFP-C2 expression vector in order to obtain EGFP-Little ROCK fusion protein constructs with Little ROCK in frame with the C-terminus of EGFP. We amplified using cDNALTF and cDNACMR primers and cloned a fragment of Little ROCK cDNA, including the entire coding sequence and part of both the 5' and the 3' UTRs into the pCR4-TOPO vector (Invitrogen). A clone of *Little ROCK *was obtained and we amplified a fragment by using the primers LTGFPF (carrying the *Eco*RI consensus sequence at 5' end) and M13F that is part of pCR4-TOPO vector sequence and downstream the *Eco*RI restriction site. The PCR product was digested with *Eco*RI and ligated into the pEGFP-C2 expression vector at *Eco*RI cloning sites. Sequencing reactions confirmed that the nucleotide sequence of *Little ROCK *was in frame with EGFP. We isolated the *Little ROCK *clone showing the A662/C667 nucleotides and we generated the *Little ROCK *clone carrying G662/T667 nucleotides by using the Stratagene (Stratagene, Cedar Creek, TX) site-directed mutagenesis quick change mutagenesis kit and primers MU2LTF and MU2LTR (Table 1). The two clones were respectively named pEGFP-LRAC and pEGFP-LRGT.

### Cell Transfection and Western Blotting

HeLa cells were cultivated in growth medium (10% FBS, 1× Penicillin/streptomycin, DMEM) until 60-80% confluent. Transfection reactions were carried out with PolyFect reagent (Qiagen, Valencia, CA) using a DNA/PolyFect ratio of 1 (μg)/10 (μl) following the manufacturer's instructions. The cells were washed twice with 1× PBS 48 hours after transfection and fresh medium containing 10 μM of MG132 or DMSO (same solvent used to dissolve MG132) as a control was added. The cells were incubated at 37° for 4 hours. Protein lysates were prepared in lysis buffer (50 mM Tris pH 7.5, 150 mM NaCl, 5% Glycerol, 1% Triton, 10 mM MgCl_2_, 1 mM EGTA, 1 mM DTT, 25 mM NaF, 20 mM b-Glycerophosphate, 1 mM Na_3_VO_4_, 2 mM PMSF, 1× Protein Inhibitors cocktail). After protein quantification by BCA protein assay (Pierce, Rockford, IL), 50 μg of protein lysates were loaded onto a 12% SDS page. Protein was then transferred onto a nitrocellulose membrane and then treated with blocking solution (1× TBST, 5% skim milk). The mouse anti-GFP antibody (Covance Inc, Princeton NJ, USA), was diluted 1:2000 in 1× TBST and 2% skim milk. The secondary Goat anti-mouse HRP conjugated antibody (Santa Cruz Biotechnology, Santa Cruz, CA) was diluted 1:10000 in 1× TBST and 2% skim milk. ECL plus western blotting detection system (GE Healthcare, Bukinghamshire, UK) was used for protein detection. The blotted membranes were then analyzed on a Typhoon scanner and the protein band intensity was measured by ImageQuant TL software (GE Healthcare). Differences in protein abundance were compared by t-test.

## Authors' contributions

MCM carried out the bio-informatic analyses, molecular genetic studies, expression studies, recombinant protein analysis and drafted the manuscript. CDB and KM performed expression studies, plasmid cloning and recombinant protein studies. SER provided Heart SCORE cohort and advice in analysis. HS and MEM participated in the design of the study, provided important study reagents and techniques, and helped to draft the manuscript. GSH participated in the design of the study, performed the statistical analysis, conceived of the study, and participated in its design and coordination and helped to draft the manuscript. All authors read and approved the final manuscript.
